# Editorial: Women in cancer immunity and immunotherapy

**DOI:** 10.3389/fimmu.2024.1466010

**Published:** 2024-08-21

**Authors:** Min Yao, Evelyn Ullrich, Adriana Albini

**Affiliations:** ^1^ Department of Immunology, Medical School, Nantong University, Nantong, China; ^2^ Goethe University Frankfurt, Department of Pediatrics, Experimental Immunology and Cell Therapy, Frankfurt am Main, Germany; ^3^ Frankfurt Cancer Institute, Goethe University, Frankfurt am Main, Germany; ^4^ Scientific Directorate, Eurpean Institute of Oncology (IEO) Scientific Institute for Research, Hospitalization and Healthcare (IRCCS), Milan, Italy

**Keywords:** immunity, immunotherapy, immunology, inflammation, cancer, infections, vaccines, women

Marie Skłodowska-Curie was the first woman to receive a Nobel Prize, in 1903, and the only woman to receive two Nobel prizes, the second in 1911. She once said, “It is better to enrich oneself with knowledge than to adorn oneself with jewelry.” This sentiment undoubtedly resonates with women scientists around the world. 120 years later, Katalin Karikó, another science role model won the Nobel Prize in Physiology or Medicine 2023 for her discovery related to immunology. Katalin Karikó as well as Drew Weissman discovered that modifications of RNA prevented unwanted inflammatory reactions and increased the production of desired proteins. The discovery laid the foundation for effective mRNA vaccines.

Still, a lower percentage of women scientists globally have a successful career, particularly in medical research. Although progress has been made, significant steps are still needed to bridge the gender gap, especially in leadership roles. In the field of life sciences women are now at parity or even overrepresented at degree-granting stages. However, further along the line, balancing family responsibilities with demanding scientific careers, along with limited access to professional networks and mentorship, creates increasing barriers. The “leaky pipeline” phenomenon describes the disproportionate loss of women and other underrepresented groups as they advance through their careers.

A study published in 2020 (https://www.pnas.org/doi/10.1073/pnas.1914221117) reveals significant gender disparities in total productivity and impact among scientists since 1955, despite men and women having comparable annual publication rates and performance. Analyzing over 7 million scientists’ careers, the findings show that male scientists publish an average of 13.2 papers during their careers, compared to 9.6 papers for female scientists. High-impact male authors also receive 36% more citations than their female counterparts, highlighting substantial gender gaps in career-wide productivity and impact. These disparities are largely due to differences in career length and dropout rates, with female scientists facing a 19.5% higher risk of leaving academia each year compared to their male counterparts.

Historically, women have played a pivotal role in the field of immunology and immunotherapy, contributing important discoveries and challenging traditional scientific paradigms. Their achievements were recognized by such institutions as the American Association of Immunologists (AAI), which, unlike many scientific societies of the time, welcomed women from its inception in 1913. Martha Wollstein was one of the first female members in 1918. She conducted groundbreaking research in bacteriology particularly focusing on the study of antibodies and the immune response. Another member, Jessie Marmorston (AAI 1932), a professor of experimental medicine at the University of Southern California, made substantial contributions to immunology, endocrinology, and the study of aging. Her research focused on hormonal mechanisms and their implications for treating various diseases such as cancer and heart disease, as well as conditions associated with aging. She also leveraged her family connections with Hollywood in the 1950s to raise funds for research and likely served as a public role model for many aspiring female scientists.

Women pioneers notwithstanding, the gender gap in this field is still very wide. According to Research.com’s third edition ranking of top immunology scientists, based on the D-index (Discipline H-index) the first position occupied by a woman is at number 67, with Arlene H. Sharpe, from Harvard University. The second ranking woman is Laurence Zitvogel University of Paris-Saclay, France, at 82, and it is not until the hundreds that women appear more regularly.

It is therefore a great honor to contribute to this inaugural column dedicated to women scientists. We are deeply grateful to Frontiers in Immunology for their attention and support of our community. Following rigorous peer review by experts in the field, nine outstanding contributions have been accepted: five original research articles, three reviews, and one case report, 7 of those have first or last or both female authors and two with prominent women co-authorships. These contributions highlight the academic and research prowess of women scientists in the fields of cancer immunity and immunotherapy.

These original research articles on cancer immunity present highly valuable studies. Pancreatic ductal adenocarcinoma (PDAC) has a poor prognosis, often due to peritoneal dissemination. Exosomal ROR1 (exo-ROR1) in peritoneal fluid (PF) has emerged as a potential diagnostic and prognostic biomarker. Mittelstädt et al. analyzed exo-ROR1 in PF and plasma from various patient groups, finding the highest levels in PDAC patients with peritoneal dissemination (PER+). High exo-ROR1 levels in PF are linked to lower overall survival. Exo-ROR1 may help distinguish between non-cancerous conditions, localized PDAC (PER-), and peritoneal disseminated PDAC (PER+), offering insights for future PDAC treatment strategies.

Addressing the significant public health issue of high-risk HPV infections, Pagni et al. developed the gDE7 vaccine by combining HSV-1 glycoprotein D with HPV-16 E7 oncoprotein to treat HPV-related tumors. Despite promising results in mice, combined therapies may enhance antitumor responses by addressing immunosuppressive mechanisms involving IDO and IL-6. Studies showed that IDO inhibitors or IL-6 knockout mice improved the efficacy of gDE7 vaccines, leading to total tumor rejection in some cases. This study suggests that targeting IL-6 and IDO can enhance immune responses, offering new strategies for HPV-related tumor immunotherapy.

In addition to mechanistic studies and vaccine development, there were also studies focused on improving immunotherapy methodologies. Calcium electroporation (CaEP) facilitates cellular uptake of high Ca^2+^ concentrations, inducing cell death. While clinical trials have assessed CaEP’s efficacy, preclinical studies are needed to clarify its mechanisms. Lisec et al. compared CaEP’s efficiency with electrochemotherapy (ECT) and IL-12 gene electrotransfer (GET) in two tumor models, hypothesizing IL-12 enhances local therapies. They evaluated CaEP *in vitro* and *in vivo* using B16-F10 melanoma and 4T1 mammary carcinoma, alongside ECT. Different Ca^2+^ concentrations alone or with IL-12 GET were tested. They analyzed the tumor microenvironment via immunofluorescence, and reported that CaEP and ECT reduced cell viability dose-dependently *in vitro*, similarly effective across cell lines. *In vivo*, CaEP delayed 4T1 tumor growth by over 30 days with 250 mM Ca, comparable to ECT. IL-12 GET post-CaEP prolonged survival in B16-F10 mice but not 4T1. CaEP with IL-12 GET altered immune cells and vasculature, was more effective against 4T1 tumors *in vivo*, potentially due to immune system involvement. Combining CaEP or ECT with IL-12 GET enhanced antitumor effects, varying by tumor immunogenicity. Multimodal treatment approaches, such as radio-immunotherapy, require optimized regimens and understanding interactions between different modalities. The work presented by Remic et al. aimed to determine the optimal combination of radiation therapy with a tumor cell-based vaccine and explore the immune response to this treatment. Using B16F10 melanoma and CT26 colorectal carcinoma models, different radiation and vaccination regimens were compared. Local immune responses were assessed by evaluating immune cell infiltration at the vaccination site and within tumors. Systemic responses were evaluated by assessing tumor-specific effector cells in draining lymph nodes. The results identified that the most effective treatment was a combination of 5× 5 Gy radiation with single-dose vaccination (B16F10) or multi-dose vaccination (CT26). This approach induced local immune responses in both tumor models and significantly generated tumor-specific effector cells in draining lymph nodes of B16F10, albeit less so in CT26 models. Optimized multimodal treatment demonstrated that the vaccine induces immune responses and enhances the efficacy of tumor radiation therapy, influenced by tumor immunogenicity. James et al. highlighted the need for further research on chemotherapy’s impact on the tumor immune microenvironment (TIME) in ovarian cancer, focusing on recurrence and chemoresistance. They found that neoadjuvant chemotherapy (NACT) increases immune infiltrate and pro-tumorigenic pathways, particularly genes linked to ATF3 and EGR1, with notable upregulation in platinum-resistant cells. The study also revealed correlations between post-NACT immune parameters and platinum-free interval (PFI), suggesting potential biomarkers for chemotherapy response and resistance, which could aid in developing novel immunotherapies to combat chemoresistance.

The reviews by Ibis et al. and Li et al. both discuss the application of immune checkpoints in cancer therapy. The former explored mechanisms that immune checkpoint inhibitors (ICI) induce various types of immune-related adverse events (irAEs) by inhibiting receptors that maintain peripheral tolerance through preventing activation of autoimmune cells. They also highlighted recent research on ICI therapy in cancer and patients with pre-existing autoimmune diseases (AD). The latter reviewed the role of immunotherapy in HCC, noting limited response of HCC to monotherapy with ICIs, prompting increased research into potent anti-tumor immune strategies to potentially enhance efficacy in HCC immunotherapy. Carlini et al. analyzed and summarized the multifaceted nature of IL-10. IL-10 is a versatile cytokine central to inflammation regulation and cellular balance, primarily acting as an anti-inflammatory agent via Jak1/Tyk2 and STAT3 pathways. It also exhibits immunostimulatory properties in specific contexts, influencing conditions like cancer and infectious diseases uch as COVID-19. IL-10’s role extended to predicting severity in SARS-CoV-2 infections, where it served as an endogenous signal against hyperinflammation, suggesting potential for pharmacological strategies and natural compounds to modulate its effects beneficially.

The final case reported by Luo et al. showed us that ovarian cancer patients with multidrug-resistant might still derive the long-term benefit of the PD-1 inhibitor, even if PD-L1 is negative. HLA-B44 supertype might be the potential predictor for immunotherapy in OCCC.

Currently, women make up only about 30% of researchers worldwide. The platform created by Frontiers in Immunology specifically for women aims to encourage more women to pursue careers in scientific research and contribute to the advancement of humanity.

